# Single-incision port robot-assisted surgery for thymic carcinoid tumor resection

**DOI:** 10.1186/s13019-022-01847-1

**Published:** 2022-05-03

**Authors:** Hiroaki Shidei, Shota Mitsuboshi, Tomoko Yamamoto, Masato Kanzaki

**Affiliations:** 1 Department of Thoracic Surgery, School of Medicine, Tokyo Women’s Medical University, 8-1 Kawada-cho, Shinjuku-ku, Tokyo, 162-8666 Japan; 2 Department of Surgical Pathology, School of Medicine, Tokyo Women’s Medical University, 8-1 Kawada-cho, Shinjuku-ku, Tokyo, 162-8666 Japan

**Keywords:** Robot-assisted thoracoscopic surgery, Single-incision, Mediastinal tumor, Thymic carcinoid

## Abstract

**Background:**

Multiple endocrine neoplasia (MEN) is divided into MEN type 1 (MEN-1) and MEN type 2 (MEN-2). MEN-1 may be associated with thymic carcinoid tumors. We present a case of the surgical removal of a thymic carcinoid associated with MEN-1 via a single-incision port RATS.

**Case presentation:**

A 39-year-old male patient with multiple endocrine neoplasia type 1 (MEN-1) who had an anterior mediastinal mass was referred to our hospital. The patient had undergone total parathyroidectomy and auto-transplantation of a partial parathyroid for hyperparathyroidism 6 years ago. Chest computed tomography revealed an isolated anterior mediastinal mass on the thymic gland with a maximum diameter measuring 22 mm. Thymic carcinoid tumor is classified as MEN-1 and has a poor prognosis, so we decided to remove the tumor. Single-incision port RATS was performed, and thymic carcinoid was confirmed in pathology.

**Conclusions:**

This report demonstrates that thymic carcinoid tumor removal is feasible and easy to perform via single-incision port RATS.

## Background

Multiple endocrine neoplasia (MEN) is divided into MEN type 1 (MEN-1) and MEN type 2 (MEN-2). MEN-1 may be associated with thymic carcinoid tumors [1, 2]. Mediastinal tumors (MTs) are usually resected by video-assisted thoracoscopic surgery [3]. Since 2018, when robot-assisted thoracoscopic surgery (RATS) became covered by Japan’s National Health Insurance, many patients with MTs have undergone resection by RATS [4]. Although RATS has various approaches, a skin incision is required for each port insertion. The most favorable advantage of RATS is the greater range of motion of the joint-equipped robotic forceps that makes procedures to be performed deep in the thoracic cavity much easier than conventional thoracoscopic surgery under three-dimensional high vision. The increased surgical accuracy also facilitates the performance of complex procedures. Here, we present a case of the surgical removal of a thymic carcinoid associated with MEN-1 via a single-incision port RATS.

## Case presentation

A 39-year-old male patient with MEN-1 who had an anterior mediastinal mass was referred to our hospital. The patient had undergone total parathyroidectomy and auto-transplantation of a partial parathyroid for hyperparathyroidism 6 years ago. His vital signs showed no abnormalities. He had a temperature of 37.2 °C, blood pressure of 117/72 mmHg, heart rate of 100 bpm, respiratory rate of 16 breaths per minute, and oxygen saturation of 98% in room air. Laboratory data revealed a serum antiacetylcholine receptor binding antibody level below 0.2 nmol/L (normal, below 0.2 nmol/L), serum cancer antigen level 5 U/mL (normal, below 35 U/mL), serum α-fetoprotein level 3 ng/mL (normal, below 10 ng/mL), serum human chorionic gonadotropin β subunit level below 0.5 mIU/mL (normal, below 0.5 mIU/mL), and serum soluble interleukin-2 receptor level 292 U/mL (normal, below 475 U/mL).

**Fig. 1:**
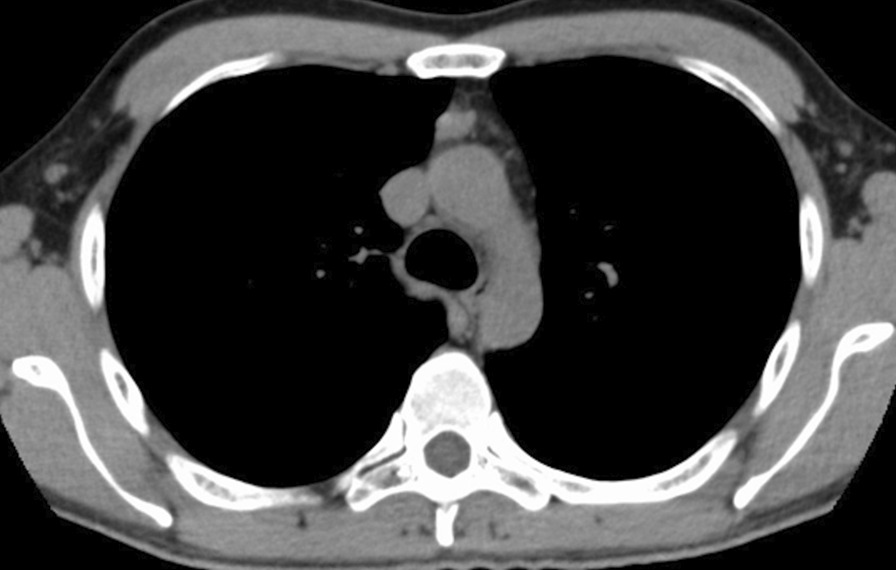
Computed tomography imaging. Chest computed tomography revealed an isolated anterior mediastinal mass with a maximum diameter measuring 22 mm without invasion to the surrounding tissues

**Fig. 2:**
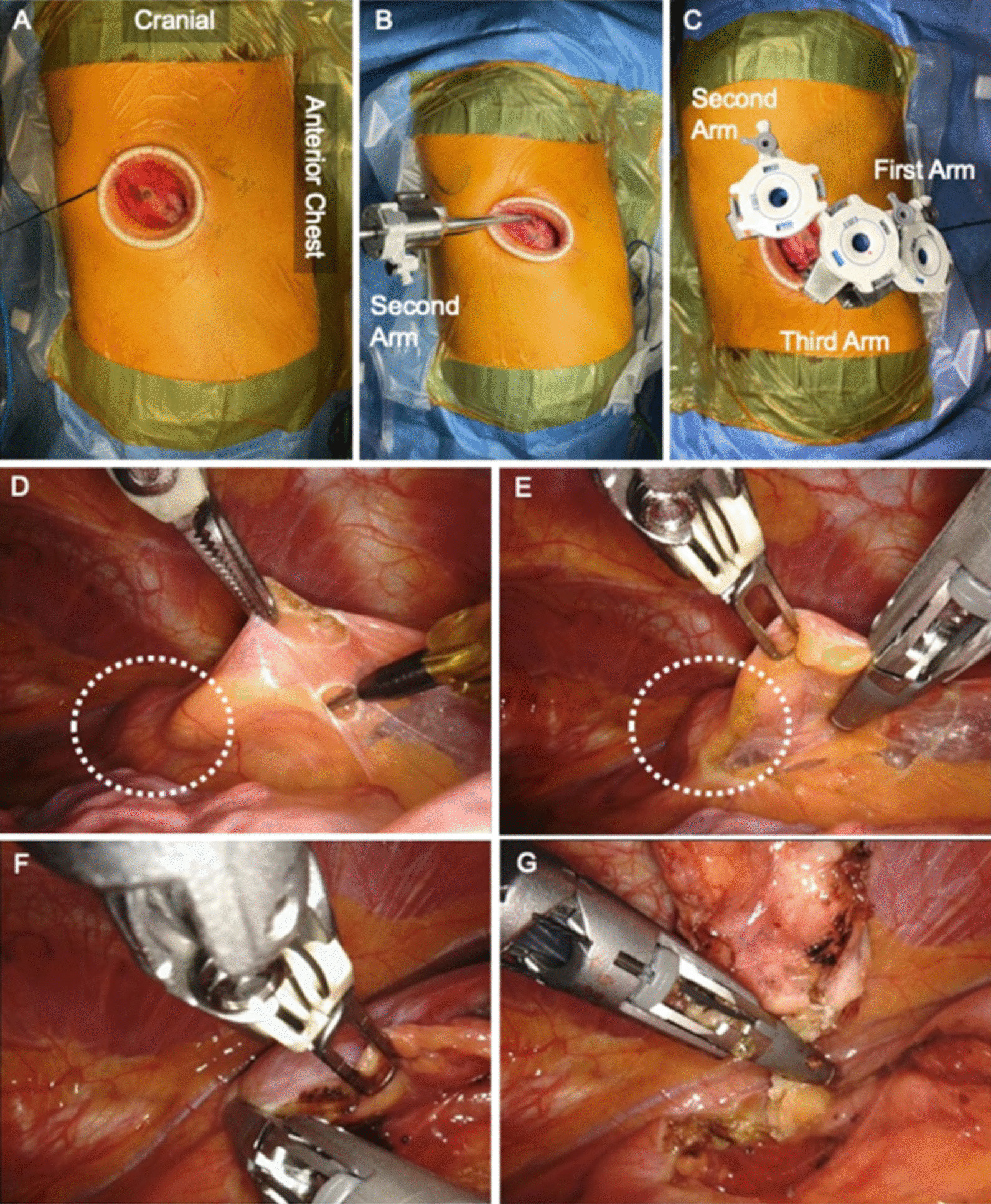
Intraoperative images. **A** A 5-cm skin incision was made at the fifth intercostal space (ICS) in the anterior axillary line, and a wound protector was placed at the subcutaneous muscular layer. **B** An 8-mm camera port placed at the fifth ICS in the anterior axillary line as the second arm. **C** The distance between each port was about 3 cm, with an 8-mm robotic port inserted each at the fifth ICS on the anterior axillary line as the second arm, at the fourth ICS on the anterior side as the first arm, and at the sixth ICS on the anterior side as the third arm. **D** The tumor is indicated by the white dotted circle. **E** and **F** The monopolar spatula attached to the 3rd arm was replaced with a da Vinci® Vessel Sealer Extend to continue the excision. **G** Complete dissection and removal of the tumor from the cranial side

**Fig. 3:**
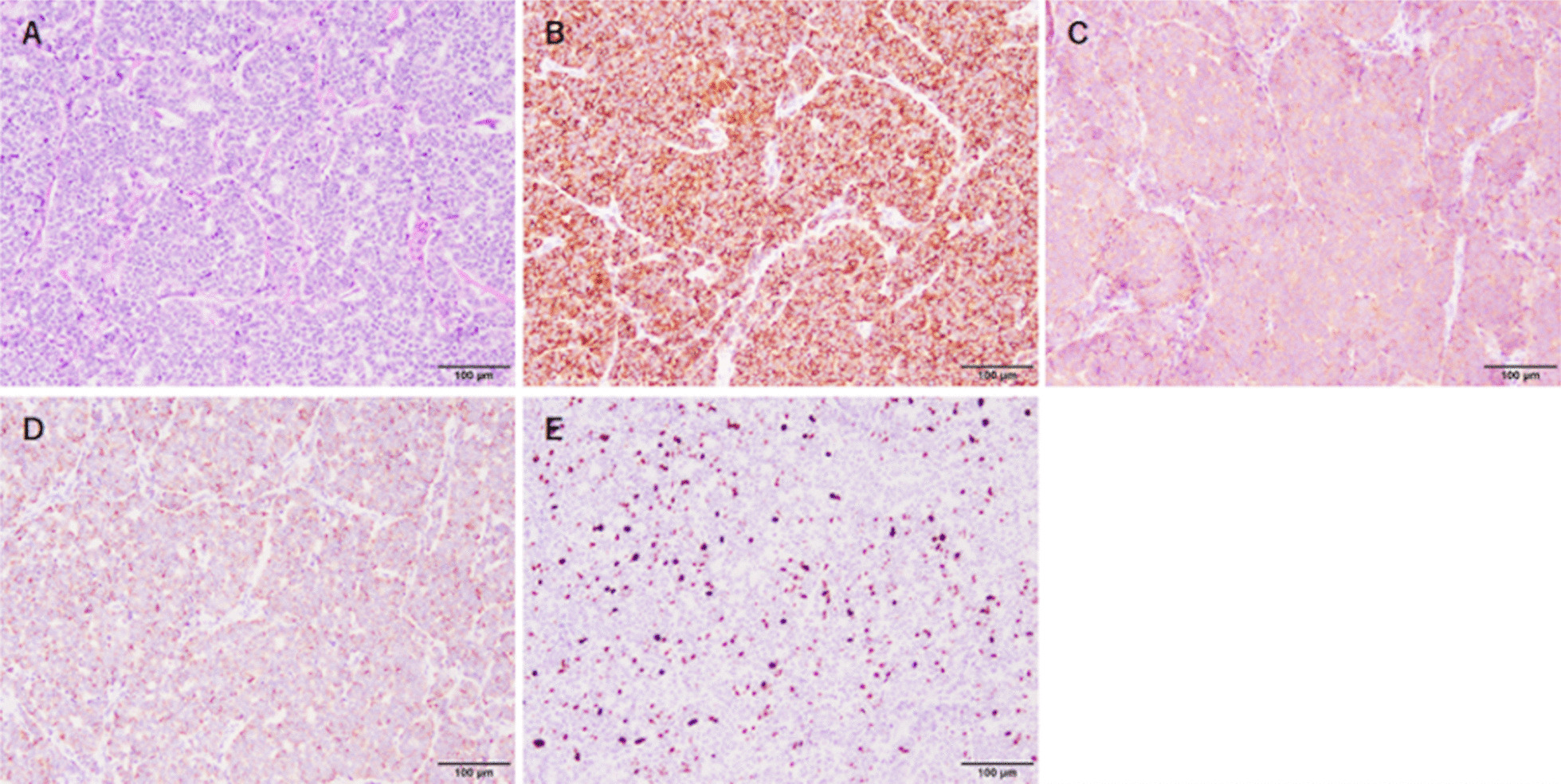
Pathological findings. **A** Hematoxylin and eosin staining revealed the presence of atypical pleomorphic cells. **B**–**E** Immunohistochemical staining was positive for **B** chromogranin A and **C** synaptophysin and weakly positive for **D** cytokeratin AE1/3 and **E** Ki-67. Scale bars, 100 μm

Chest computed tomography revealed an isolated anterior mediastinal mass on the thymic gland with a maximum diameter measuring 22 mm and without invasion into the surrounding tissues (Fig. 1). Thymic carcinoid is classified as MEN-1 and has a poor prognosis; thus, we decided to remove the tumor. The patient was intubated with a double-lumen endotracheal tube for one-lung ventilation under general anesthesia and positioned in the left lateral decubitus position. A 5-cm skin incision was made at the fifth intercostal space (ICS) in the anterior axillary line, and a wound protector (Gel POINT Mini Advanced Access Platform, Applied Medical, Rancho Santa Margarita, CA, USA) was placed at the subcutaneous muscular layer (Fig. 2A). Through the wound protector, an 8-mm camera port was placed at the fifth ICS in the anterior axillary line as the second arm. Two 8-mm assisted ports were inserted at the fourth and sixth ICS on the anterior side as the first and third arms, respectively (Fig. 2B, C). The distance between each port was about 3 cm. After the da Vinci® Xi (Intuitive Surgical, Sunnyvale, CA, USA) was positioned; a robotic arm was mounted to each port with a bipolar fenestrated grasping forceps on the first arm and a monopolar spatula on the third arm. The rigid 30° oblique viewing endoscope was used. Carbon dioxide (CO_2_) was insufflated at a set pressure of 5 mmHg. Depending on the situation, the robotic instruments on the left and right arms were replaced and da Vinci® Vessel Sealer Extend (Intuitive Surgical) instruments were also used. Part of the thymic and pericardial fat including the tumor was dissected from the inferior side, and complete dissection was performed from the cranial side, resulting in the removal of the tumor. To prevent robotic arm collisions, the left and right forceps were moved vertically with the endoscope in between (Fig. 2D–G). The final histopathologic examination diagnosed the tumor as a thymic carcinoid tumor (Fig. 3). Hematoxylin and eosin staining revealed the presence of a number of atypical pleomorphic cells. Immunohistochemical staining showed the presence of neuroendocrine markers chromogranin A, synaptophysin, cytokeratin AE1/3, and Ki-67 (5–10%) (Fig. 3A–E). Thus, confirmation of a thymic carcinoid tumor was obtained. The postoperative course was uneventful.

## Discussion and conclusions

Thymic carcinoid tumors are usually associated with MEN-1 [1, 2]. Thymic carcinoid tumors are rarer than thymomas, have a poor prognosis, and have a characteristic tendency to recur and metastasize years after the initial diagnosis and treatment. The effect of chemotherapy is unknown, but in most cases, initial surgical intervention and radiotherapy seem to help.

RATS is gaining popularity due to the many benefits offered by robotic surgical systems. As a result, RATS procedures have seen a rapid increase in Japan. The dedicated robotic instruments of the robotic surgical system are capable of motions with 7 degrees of freedom under three-dimensional view. Even in a mediastinum packed with intertwined organs, the robotic surgery system can execute surgical maneuvers smoothly and naturally [5]. RATS have gained importance for MT surgeries and have been shown to achieve results comparable to those of the video-assisted thoracoscopic surgical (VATS) approach or open surgery. Furthermore, excellent perioperative outcomes of robotic resections of malignant tumors in the anterior mediastinum have been reported [4]. Conventionally, in MT resection by RATS, a skin incision is made at each port insertion site placed strategically to avoid robotic arm collisions. In this procedure, the left and right forceps operations were moved vertically with the endoscope in between.

Ishikawa et al. [6] reported a completely endoscopic single port robotic surgery. In their procedure, a robotic endoscope and two robotic instruments were inserted through one port about 2.5-cm long at the third intercostal space on the right anterior chest. However, although they attempted pneumothorax by CO_2_ insufflation to maintain a sufficient distance from the tumor, they were unable to maintain an airtight environment. Their technique of employing cross-arm technology was useful in safely preventing collisions and achieved good clinical and excellent cosmetic results. Since the mediastinum is a tightly packed organ with a narrow space, the working space of robotic instruments needs to be expanded. Thus, CO_2_ insufflation is helpful in expanding the working space, and our technique makes this step easy. Subsequently, the robotic instruments were operated the same as in a normal RATS operation without using cross-arm technology.

This case report had some important limitations: First, although some authors agree on the use of aggressive attempts similar to the approach for thymoma, in this case, a part of the thymic and pericardial fat including the tumor was resected. Second, the indication of this single-incision total port RATS may be controversial. This procedure may require extensive experience of RATS when manipulating a wide range of operative fields such as thymectomy or when removing larger tumors. Furthermore, skilled surgeons may have been able to perform the surgery via uniport VATS. Nevertheless, the procedure outlined in this report is feasible, easy to perform, and provides dexterity during the procedure. Furthermore, since a new robotic surgery system via a single port has already been reported, this procedure may be easier to perform.

This report demonstrates that thymic carcinoid tumor removal is feasible and easy to perform via single-incision port RATS. Since the mediastinum is a tightly packed organ with a narrow surgical field, CO_2_ insufflation was performed, and an airtight environment was maintained by inserting ports into three different intercostal spaces through wound protectors. Our case thus provides further confirmation that robotic-assisted surgical systems can be successfully used for surgical intervention in MTs, given the complete resection, and uneventful postoperative course achieved in this case.

## Data Availability

All the data and materials supporting our findings are included within the article.

## Abbreviations

MEN: Multiple endocrine neoplasia
MEN-1: Multiple endocrine neoplasia type 1
MEN-2: Multiple endocrine neoplasia type 2
MT: Mediastinal tumor
RATS: Robot-assisted thoracoscopic surgery
ICS: Intercostal space
CO_2_: Carbon dioxide
VATS: Video-assisted thoracoscopic surgery
